# Animal Models as Foundational Tools in Preclinical Orthopedic Implant Research

**DOI:** 10.3390/biomedicines13102468

**Published:** 2025-10-10

**Authors:** Renata Maria Varut, Diana-Maria Trasca, George Alin Stoica, Carmen Sirbulet, Cristian Cosmin Arsenie, Cristina Popescu

**Affiliations:** 1 Research Methodology Department, Faculty of Pharmacy, University of Medicine and Pharmacy of Craiova, 200349 Craiova, Romania; renata.varut@umfcv.ro; 2 Department of Internal Medicine, University of Medicine and Pharmacy of Craiova, 200349 Craiova, Romania; 3 Department of Pediatric Surgery, Faculty of Medicine, University of Medicine and Pharmacy of Craiova, 200349 Craiova, Romania; 4 ENT Doctor Department of Anatomy, University of Medicine and Pharmacy, Discipline of Anatomy, 200349 Craiova, Romania; carmen.sirbulet@umfcv.ro (C.S.); arsenie_cristian@yahoo.com (C.C.A.); cristina.popescu@umfcv.ro (C.P.)

**Keywords:** orthopedic implants, animal models, osseointegration, biocomposites, bone regeneration, preclinical testing, biomechanical analysis, histomorphometry, micro-CT imaging

## Abstract

Orthopedic implants have a critical role in modern medical practice, being useful in bone regeneration, joint arthroplasty, and healing fractures. The success of osseointegration depends on implant properties (composition, stability, geometry, biocompatibility) and host factors (local reactivity, comorbidities). Preclinical evaluation in animal models is essential before clinical application. In orthopedic implantology, the selection and real utility of a range of animals are important, with an emphasis placed on bone–implant interface, biomechanical function, and long-term integration. Smaller animals such as rabbits and rats have widespread use in early biocompatibility and osseointegration testing, but larger animals such as pigs, sheep, and canines have a larger physiological bone similarity and can, therefore, be utilized for bearing loads in testing. Considering the utility and disadvantages of certain species—including suitability for new biomaterials, coatings, and biomechanical function—this article discusses testing methodologies such as push-out/pull-out tests, histomorphometry, and micro-CT and their utility in testing the integration of implants and regeneration of bone. Conclusions confirm a multi-species model in use in preclinical testing for the development of implants and improvements in clinical success. Unlike previous reviews, this article emphasizes translational strategies, integrates ethical perspectives in model selection, and discusses the synergistic use of imaging modalities with biomechanical tests for comprehensive assessment.

## 1. Introduction

Orthopedic implants represent a pillar in the treatment of fractures, degenerative disease, arthroplasties of joints, and bone regeneration operations. There have been significant recent advances in the technology of materials used in implantology, with a series of advanced biomaterials—including metallic, ceramic, polymeric, and composite systems—being developed to improve osseointegration, durability, and compatibility with the human body. Osseointegration, the direct connection between bone and implant surface, is a critical factor for implant durability. Material composition, implant geometry, surface characteristics, and local tissue response all influence this process [1]. Animal models are essential in preclinical research, as they simulate material behavior in living tissues under physiological conditions. They allow for the examination of bone–implant healing and integration in environments that mimic mechanical loading and systemic influences. These models offer the best physiological mimic of human conditions before graduating to the clinical trial setting. The choice of animal model in orthopedic research depends on the body size and weight of the animal, the similarity of its bone structure to that of humans, and the ethical issues raised by its use. The choice of animal species is also influenced by other factors such as the ease of handling the animal, the availability of the species, and their suitability for use in standardized environments and procedures. Before selecting an animal species, selection tests are often applied. Although no animal model perfectly mimics human bone physiology, different species are used to answer different research questions, each with advantages and limitations to its use [2].

In this review, the term biocomposite refers specifically to hybrid materials combining biological components (e.g., bone, collagen, stem cells) with synthetic or natural biomaterials designed to enhance bone healing and regeneration. These biocomposites are used to help with healing or to replace bone tissue that has been damaged or lost through injury, disease, or surgery. The materials usually employed in these biocomposites include hydroxyapatite (HA), calcium phosphate (CaP), bioactive glasses, and titanium alloys, all of which have been found to have great potential in enhancing the performance of the implant. These biocomposites are used in various orthopedic surgeries, including joint replacement, implanting prostheses, and the management of fractures. They have two major objectives of improving healing and preventing complications and improving patient living standards.

Research on current topics in orthopedic and regenerative medicine, such as the application of biocomposites in bone regeneration, is quite interesting and relevant. An orthopedic implant is a medical device used to replace all or part of a bone structure in the body, in order to restore its functional and mechanical purpose. Among the elderly population, people who experienced traumatic injuries, or those with chronic medical conditions, the demand for orthopedic implants is very high. While there are currently many basic materials with functionalization techniques and processes to obtain them, the field of implant materials is being researched assiduously. However, most metallic implants have been found to be incompatible and poorly osteogenic. The surface chemistry and energy, roughness, and crystallinity are some of the properties that have been reported to enhance or depress implant performance [3]. In this review, we will discuss the types of biocomposites used in orthopedic implants, the design parameters, and the most common animal models used to test implants before starting clinical trials. Moreover, the review will highlight the biomechanical and biological assessment protocols used to determine implant efficacy, the limitations of the current animal models, and the new trends in implantology research. The novelty of our work lies in bridging preclinical animal research with translational strategies relevant for human applications, integrating ethical considerations in species choice, and discussing how the combination of imaging modalities with biomechanical tests provides a more robust evaluation framework. 

## 2. Animal Models for Studying Orthopedic Implants

Orthopedic implants are generally categorized as metallic, ceramic, polymeric, and composite biocomposites, each engineered to meet specific mechanical and biological requirements in clinical application. Metallic implants, particularly titanium alloys, are preferred for load-bearing applications such as joint replacements due to their excellent biocompatibility, high strength, and corrosion resistance. Ceramic materials, including hydroxyapatite and bioactive glasses, are osteoconductive and are commonly used as coatings or scaffolds to enhance bone regeneration. Polymeric materials such as PLA and PEEK offer distinct advantages: PLA is biodegradable and used in resorbable fixation devices, whereas PEEK provides durability and biocompatibility, making it suitable for load-sharing implants. Cermet and ceramic–matrix composites combine the advantages of ceramics and polymers, providing the required levels of bioactivity and mechanical performance for bone fillers and fixation devices. Recently, magnesium-based composites have gained attention as promising candidates for biodegradable implants in fracture fixation [4,5,6]. Surface modifications, including nano-coatings, further enhance implant performance by accelerating osseointegration and reducing healing time. Collectively, these materials and techniques support the development of tailored solutions for diverse orthopedic challenges, from pediatric to complex reconstructive cases, aiming to improve bone integration and long-term clinical outcomes [7,8,9,10]. The reference standard is human bone with complete osteonal remodeling, slow healing and high tissue integration. Other non-human primates also exhibit similar remodeling and vascular supply, but raise enormous ethical concerns. Rodents are inexpensive and easy to handle, but their limited osteonal remodeling and accelerated healing make them unsuitable for cortical bone research. Rabbits and dogs show moderate-to-high healing and tissue integration, though ethical concerns vary. Pigs and small ruminants (sheep, goats) closely resemble human bone remodeling and vascularity, with outcomes influenced by factors such as age and season. These species are selected based on the specific requirements of orthopedic research, balancing biological relevance and ethical considerations. Vascularization characteristics vary across species and strongly influence implant healing outcomes. In humans, blood supply varies considerably by anatomical site, which partly explains site-specific differences in osseointegration. Non-human primates display vascularization patterns very similar to humans, enhancing their translational relevance, although their use is ethically challenging. Rodents, by contrast, exhibit minimal cortical vascularity, limiting their value in cortical bone studies despite their convenience and low cost. Rabbits provide a rapid vascular response in young animals, which facilitates efficient assessment of short- to medium-term healing, but complicates extrapolation to long-term human outcomes. Dogs show intense remodeling activity and a high degree of vascularization, making them suitable for dynamic osseointegration studies, though ethical concerns restrict their use. Pigs resemble humans in vascular architecture, which enhances their role in load-bearing implant research, while sheep display seasonally variable vascularization that must be considered when interpreting results. Goats, particularly in trabecular regions, exhibit dense vascular networks, supporting studies focused on cancellous bone integration (Table 1). Taken together, these interspecies differences in vascularity are critical for selecting appropriate animal models to evaluate implant osseointegration under biologically relevant conditions [11,12,13].

Animal models are essential for understanding the biological behavior and mechanical performance of orthopedic implants. They allow researchers to investigate osseointegration, biomechanical stability, and implant degradation under physiological relevant conditions. Below is a discussion of the most commonly employed animal models.

### 2.1. Small Animal Models: Rodents and Rabbits

**Figure 1:**
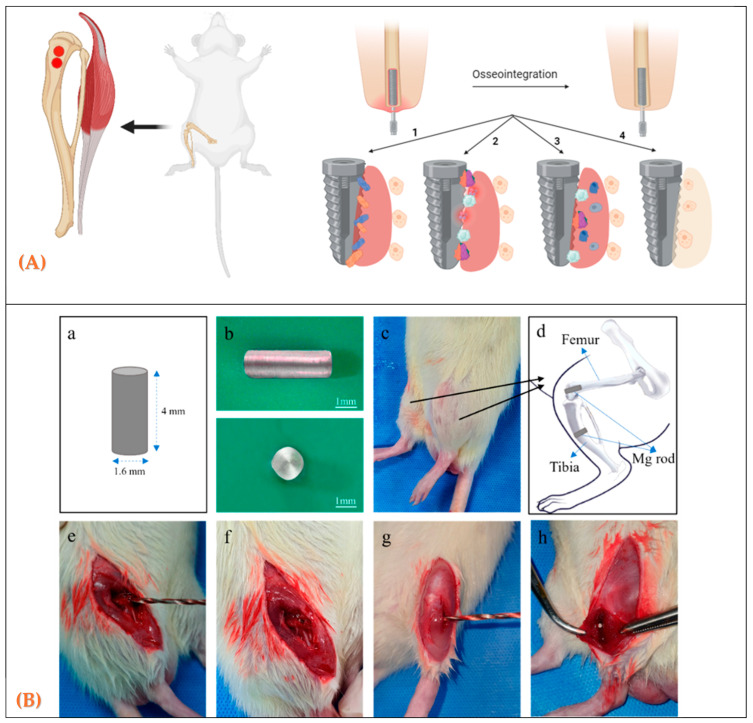
(**A**) Anatomical sites in rats used to evaluate biomaterials during bone healing. (**A**) Schematic diagram of implantation sites; (**B**) design and placement of cylindrical rod implants for in vivo testing; (**B**) (**a**–**h**) Surgical procedure for Mg rod implantation in rat tibia. (**a**) Schematic illustration of the Mg rod (4 mm × 1.6 mm). (**b**) Photographs of the Mg rod (side and top views). (**c**) Experimental animal showing the surgical area. (**d**) Schematic diagram of the implantation site and fixation method. (**e**) Exposure of femur and tibia after incision. (**f**) Preparation of the femoral condyle for drilling. (**g**) Drilling of the tibial medullary canal. (**h**) Insertion of the Mg rod into the tibial canal. [14].

Rodents (Rats and Mice): Rodents are widely used due to their low cost, ease of handling, and well-characterized genetics. They are valuable for preliminary investigations, particularly for evaluating toxicity and biocompatibility. However, their small skeletal size and the limited trabecular bone remodeling restrict their usefulness in studying long-term osseointegration (Figure 1). 

**Figure 2:**
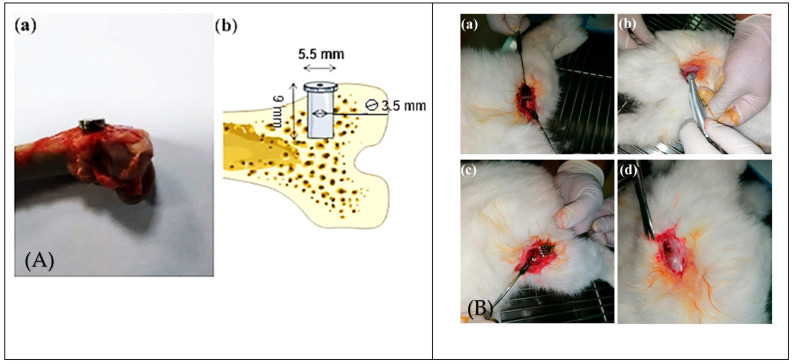
Surgical procedure and implant sites in rabbit femur models. (**A)** (**a**,**b**) Photograph and schematic representation of implant placement in bone. (**B**) Stepwise surgical sequence: (**a**) exposure of the lateral femur; (**b**) cortical drilling; (**c**) press-fit implant insertion; (**d**) wound closure. These images highlight reproducible surgical methods for evaluating implant anchorage and osseointegration in trabecular bone [18].

Rabbits: The rabbit model, previously employed in our research to investigate the osseointegration potential of 3D-printed titanium implants functionalized with lithium-doped hydroxyapatite coatings, has demonstrated a pivotal role in evaluating implant–bone interactions and mechanical stability. Because of their rapid skeletal maturity, anatomical accessibility and bone structure sharing key characteristics with human trabecular bone, rabbits are generally considered an optimal choice for initial-stage orthopedic implant studies [15]. Such attributes enable reproducible biomechanical testing, including pull-out and push-out assays, which are important for determining implant anchorage and integration. Our previous work showed that demonstrated a statistically significant improvement in bond strength between bone and functionalized implants, compared with uncoated, unfunctionalized controls, and that advanced coating technologies are effective in improving osseointegration. In this review, we contextualize the rabbit model within a comparative framework of preclinical research, emphasizing its critical role in the early-phase validation of novel implant materials and surface modifications (Figure 2). However, the rapid rate of bone turnover that is characteristic of rabbits makes it challenging to predict the long-term results that would be expected in a human clinical trial. On the other hand, due to the accelerated turnover, it is possible to efficiently evaluate the short- and medium-term results of osseointegration and thus understand the initial implant–host interaction. This is then invaluable information for advancing to subsequent studies in larger animal models, such as sheep and pigs, which are more suitable for long-term investigations [16].

By integrating findings from our prior research into the broader discourse on animal models in implantology, we highlight the relevance of the rabbit model as a foundational step in a sequential, multi-species preclinical framework. This methodology enables robust validation of implant performance across diverse biological and mechanical environments, ultimately advancing the translational potential of orthopedic implant technologies for clinical applications [17,18].

### 2.2. Large Animal Models: Sheep, Pigs, and Dogs

Sheep are frequently used due to their bone size and structure, which are close to those of humans. They are ideal for studying long-term osseointegration and mechanical stability, especially for load-bearing implants. Their use is widespread in large-scale studies involving fracture healing and joint replacement. However, seasonal variations in bone remodeling in sheep can complicate data interpretation.

Pigs, particularly miniature breeds, are used in orthopedic research due to their bone anatomy and physiology, which closely resemble those of humans. They are excellent models for studying osseointegration and implant performance in weight-bearing conditions. However, pigs’ large size can make handling and long-term care more challenging.

Despite ethical concerns regarding the use of companion animals, dogs are still used in some studies due to their bone composition, which is similar to that of humans. Their larger bones allow for the testing of human-sized implants. However, their high costs and ethical considerations limit their use [19].

### 2.3. Optimal Implantation Site for Rabbit Models: Considerations and Relevance for Human Applications

The choice of an appropriate implantation site in rabbit models depends on the specific implant design, intended function, and its potential applications in human orthopedics. Selecting an appropriate anatomical location is essential for replicating the desired biomechanical environment and ensuring that the findings translate effectively to clinical scenarios. Based on considerations for the use of the rabbit model as a substitute for human trabecular bone, several placement options have been evaluated in many studies:

- Transchondral Placement. This approach, which involves positioning the implant through the cartilage, may lead to gap formation at the interface, resulting in the potential penetration of synovial fluid from the joint space. Such penetration could alter the mechanical and biological environment of the implant–bone interface, thereby compromising implant performance and osseointegration F [18].
- Intramedullary or Intra-Diaphyseal (Axial) Placement. Positioning the implant within the intramedullary canal or along the diaphyseal axis offers extensive contact with the endosteum and bone marrow. However, due to the anatomical conical mismatch between the implant and the diaphyseal shape, this technique may result in incomplete surface contact. Hence, the lack of full bone coverage may lead to inaccurate evaluation of the implant stability and lower osseointegration values [20].
- Exclusive Intracortical Placement. Evaluating the osseointegration of an implant solely based on cortical bone contact may be inaccurate as it does not include the role of cancellous (trabecular) bone which is vital for the mechanical strength and healing capacity of implants used in load-bearing applications [21].
- Intracondylar Placement. The intracondylar placement method, which is used in many studies, makes contact with both cortical and trabecular bone. This dual interface enables the study of implant behavior under physiological strains, and thus improves the realism of the model for the simulation of human clinical conditions [22]. The lateral femoral condyle of the rabbit is of the right size and easily accessible and thus a suitable place to test implants. This position not only enables the fixation of test implants of different sizes but also offers a distinct chance to examine cortical and metaphyseal trabecular bone. Such dual contact improves the clinical relevance of this model as compared to the isolated diaphyseal placements which do not include trabecular bone in the analysis. The distal femur, especially the lateral condyle, is loaded physiologically during everyday activities including hopping, jumping and standing and thus simulates the load distribution of a bone–implant interface in humans. This characteristic is useful in assessing load transfer and implant stability in dynamic conditions. A bilateral implantation model is usually employed, where the implants are inserted into both the femoral condyles of the same animal. This technique is very efficient in reducing the number of animals used in a study and at the same time enables one to make reliable and randomized within-subject comparisons of the implants of different properties. Such a strategy is also consistent with the ethical guidelines for animal research, minimizing the number of animals used and also allowing for more powerful within-subject comparisons of the treatment effects on the study variables [23].

## 3. Biomechanical and Biological Testing of Implants

Biomechanical tests are essential for evaluating the success of orthopedic implants since they provide essential information about mechanical stability, osseointegration and long-term success. These tests reveal whether the implant can bear the mechanical loads experienced in daily life. Two of the most commonly employed biomechanical testing methods are pull-out or push-out tests, along with imaging techniques like histomorphometry and micro-computed tomography (micro-CT). The following section provides a general overview of these techniques, their applicability and the role they play in evaluating the level of osseointegration of orthopedic implants.

### 3.1. Push-Out and Pull-Out Tests

The shear forces that are generated during retention assessments subject many of the animal models used to study implant retention in bone to them. The push-out and torsion tests are frequently performed to study these mechanical stresses. However, there are some limitations of push-out tests in evaluating the force at the bone–implant interface. The shape of the implant, surface roughness and the perfect match of the implant fixation system with the force application device are the other factors which can critically affect the experimental results. Theoretically, the torsion test is better than the push-out test in that it ensures that the forces are distributed uniformly at the bone–implant interface. However, such experiments are generally more complicated and time-consuming than push-out tests.

**Figure 3:**
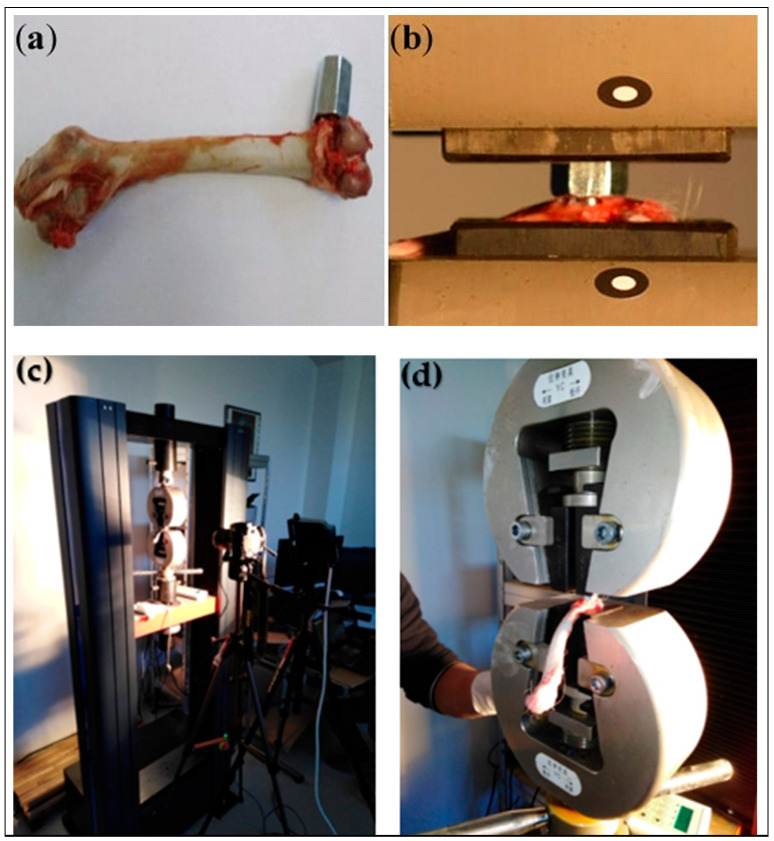
Setup for tensile pull-out testing of orthopedic implants. (**a**) Hexagonal adaptor attached to 3D titanium implant; (**b**) femur fixed with implant and adapter mounted in traction machine; (**c**) experimental device for tensile testing; (**d**) clamping support. These images demonstrate the methodology for quantifying mechanical stability and implant anchorage strength [18].

- Push-Out Tests: The shear strength of the bone–implant interface is usually determined by push-out tests that are performed by applying a controlled force perpendicular to the implant axis, measuring a force–displacement curve which quantifies the degree of osseointegration. They can be employed to determine the mechanical state ex vivo of biological fixation of the implant, usually from an animal model [24]. This particular test is very valuable in looking at the impact of different surface treatments such as porous coatings or bioactive materials which are designed to enhance the bone ingrowth and thus the stability. It should however be noted that in a research study, titanium implants were subjected to push-out testing after being coated with hydroxyapatite (HA) and it was found that the forces needed to dislodge the HA-coated implants were much higher than those for the uncoated implants. The result indicated that HA coating improved the bone–implant strength and therefore the osseointegration [23].
- Pull-Out Tests: Pull-out and push-out tests are performed in the same manner, except for the force being applied in the opposite direction, which is used to pull the implant away from the bone. This test is generally employed to determine the strength of screw-type or coated implants, where the mechanical interlocking of the implant threads with the bone is the primary means of stability [25]. Pull-out tests conducted on magnesium-based screws demonstrated that the screws provided adequate initial fixation strength for fracture healing. Over time, the screws gradually degraded, allowing for natural bone regrowth without compromising mechanical stability during the healing process (Figure 3) [26].

### 3.2. Histomorphometry

Bone histomorphometry is a quantitative analysis of undecalcified bone biopsies used to evaluate structure and remodeling. In implantology, it is considered the gold standard for assessing osseointegration, peri-implant bone formation, and the bone-to-implant contact (BIC) coefficient. Because the method is invasive and destructive, it is not suitable for long-term studies in humans. For this reason, it is employed primarily in experimental research, including animal models, such as canine mandibular and rabbit tibial studies [27].

- Bone–Implant Contact: One of the most important parameters in histomorphometry is BIC percentage, which indicates the percentage of implant surface in direct contact with the bone over the entire length of the implant. A higher BIC percentage indicates better osseointegration and greater mechanical stability. Histomorphometric analysis of titanium implants with a nano-hydroxyapatite coating revealed a significantly higher BIC percentage compared to uncoated implants, indicating that the nano-coating enhanced the implant osteointegration [28].
- Quantification of Bone Formation: Histomorphometry can also be used to quantify the amount of new bone formation around the implant. Thus, cross-sections of the bone–implant interface can be examined to determine the thickness and quality of the newly formed bone. Calcium phosphate-coated implants were investigated in numerous studies, in which histomorphometric tests showed increased peri-implant bone formation for coated compared with non-coated implants, confirming the osteoconductive nature of the coating [29].

### 3.3. Micro-CT Imaging

In recent decades, an impressive number of imaging techniques have been developed to complete analyses in the biomedical field, namely micro-computed tomography (micro-CT), scanning electron microscopy (SEM), conventional light microscopy (LM), and confocal laser scanning microscopy (CLSM).

Micro-computed tomography (micro-CT) is a miniaturized version of a CT and has a resolution in the order of 2 μm. Micro-CT works with a sealed microfocus X-ray source, a charge-coupled device camera, and a step-by-step platform that receives bone samples. The great advantage is the fact that bone biopsies or animal bone fragments can be analyzed when still in the fixative, being a fast, accurate and non-destructive imaging technique. Micro-CT is particularly valuable in longitudinal studies, where the same sample can be imaged multiple times to track changes in bone structure and implant integration over time.

- Non-Invasive Assessment of Bone Healing: Micro-CT is a useful analysis tool in implantology, allowing the visualization and evaluation of peri-implant new bone growth, and the evaluation of the recovery of fractures to normal bone stiffness and strength. In several studies, quantities such as bone volume, bone volume fraction, and mineral density in the fracture callus were measured using micro-CT. In a preclinical study, micro-CT imaging was employed to track the bone regeneration process occurring around the magnesium-based implants. The scans revealed progressive bone growth and gradual implant erosion of the implant over time, thus enabling the assessment of the implant’s performance in real time [25].
- Evaluation of Porous Implants: Micro-CT is also used to evaluate the effectiveness of porous bone replacement materials, evaluating important key factors related to the morphological properties of the implants (pore size, pore interconnectivity, anisotropy, etc.) as well as the osseointegration process (bone mineralization, bone ingrowth). By analyzing the 3D structure of the bone–implant interface, micro-CT quantifies the extent of bone infiltration into the porous regions of the implant, providing insights into how well the implant supports new bone formation. Many studies have used micro-CT to evaluate 3D-printed porous implants to visualize local cellular processes (adhesion, growth, migration, matrix synthesis, and differentiation) developed at the implant–bone interface [28].
- Implant Degradation Monitoring: Conventional implant materials are generally superior in load-bearing capacity, fatigue resistance, wear resistance, and corrosion resistance compared with those based on magnesium and its alloys. However, in this case, micro-CT is very useful in the assessment of the degradation process, whereby it can be used to quantify the corrosion pitting on the implant surface, as well as new bone formation around it. This enables researchers to observe both implant degradation and bone remodeling in response to its changes, by capturing 3D images at different time intervals. In the micro-CT imaging of magnesium implants, it was shown that the degradation time of the material is relatively long and is accompanied by the formation of new bone tissue, thus ensuring that the implant continues to provide the required mechanical support for bone growth [29].

## 4. Emerging Directions in Biomaterials and Implant Research

Recent advances in orthopedic implantology and biomaterial research have significantly expanded the translational potential of preclinical studies. Several reviews emphasize the need for rigorous biocompatibility testing and careful material selection to ensure long-term safety and clinical relevance [30,31,32]. Novel biodegradable systems have been developed to minimize device-associated infections while maintaining mechanical stability [33,34]. Likewise, magnesium- and bioactive glass-based implants demonstrate promising biodegradability and osteogenic potential in animal models, underlining the importance of tailoring degradation rates to bone healing processes [35].

The role of animal models remains central, but recent insights have highlighted their limitations and the ethical considerations associated with species selection. Updated overviews stress that species-specific vascularization, remodeling, and healing characteristics directly affect translational accuracy [36,37]. In parallel, 3D bioprinting has emerged as a transformative technology, enabling patient-specific scaffolds with precise control over porosity, architecture, and bioactivity [38,39]. Recent years have also seen a surge in the application of artificial intelligence (AI) to imaging and histomorphometric analysis, with early validation studies demonstrating that AI-assisted reconstructions can reliably quantify trabecular microarchitecture and improve analytical throughput [40,41]. Large-scale prospective evaluations of implant osseointegration, involving thousands of implants, provide strong clinical correlations for these preclinical findings [42].

A growing body of literature has also investigated bioactive coatings as a strategy to reduce infection and accelerate osseointegration. Recent systematic reviews confirm the effectiveness of both contact-killing and drug-releasing coatings in improving implant performance [43]. Complementary reviews on biomaterials used in trauma and orthopedic surgery highlight the transition from conventional materials toward nanostructured and multifunctional biomaterials that integrate antimicrobial, osteoinductive, and angiogenic properties [44,45].

Particular attention has been devoted to nanotechnology in orthopedic surgery. Nanostructured coatings, nanoparticles, and nanocomposite scaffolds improve cell adhesion and tissue integration while reducing biofilm formation [46,47]. These findings, together with ongoing progress in additive manufacturing and biomimetic material design, position the field at the threshold of a new era in which preclinical animal models increasingly integrate advanced imaging, AI-driven quantification, and smart biomaterials to bridge the gap between bench and bedside.

## 5. Methodology

This review was conducted as a narrative literature review incorporating structured search strategies to ensure the inclusion of relevant evidence. Searches were performed across PubMed, ScienceDirect, and Google Scholar using combinations of keywords such as “orthopedic implants,” “animal models,” “osseointegration,” “biomechanical testing,” “biocomposites,” and “bone regeneration.” The search strategy emphasized studies published within the last five years, while older but seminal works were also considered when they provided critical foundational insights.

Inclusion criteria targeted full-text articles in English that addressed at least one of the following aspects: (i) types and properties of orthopedic implant materials, including metals, ceramics, polymers, and composites; (ii) preclinical evaluation of implants using small and large animal models; or (iii) assessment methodologies such as histomorphometry, push-out/pull-out testing, and micro-CT imaging. Exclusion criteria were applied to conference abstracts, commentaries, editorials, and studies not directly relevant to orthopedic implantology or preclinical validation.

The aim of this narrative synthesis was to map the current evidence landscape regarding animal models in orthopedic implant research, highlighting experimental findings, methodological advances, and species-specific considerations. The focus remained on descriptive and comparative analysis rather than quantitative meta-analysis or formal risk-of-bias assessment.

## 6. Conclusions

Animal models are still essential in orthopedic implantology research, for the strict preclinical assessment of implant materials—including metals, ceramics, polymers, and composites—together with novel biocomposites designed for enhanced osseointegration, stability, and biocompatibility. As our review shows, model selection is crucial and must align with the implant’s planned application and physiological load. Initial biocompatibility screening can be performed in small animals, such as rodents, while larger models, including sheep, mimic human bone mechanics and are suitable for load-bearing experiments. Biomechanical and imaging analyses reveal that the biocomposites, including titanium alloys and magnesium-based materials, have potential to improve bone apposition and stability. Further study and optimization of animal models will advance implant development, reduce complications, and improve recovery time. Importantly, this review distinguishes itself by emphasizing translational strategies that connect preclinical testing with clinical outcomes, addressing ethical considerations in model selection, and advocating for a combined imaging-and-biomechanical approach to ensure comprehensive evaluation of implant integration. Looking ahead, several emerging technologies are expected to transform preclinical and clinical implant research. Artificial intelligence (AI) applied to imaging modalities such as micro-CT and histomorphometry can provide automated, high-throughput, and objective analysis of bone–implant interactions, enabling more precise quantification of osseointegration and implant performance. In parallel, 3D bioprinting of patient-specific scaffolds offers unprecedented opportunities for tailoring implant geometry, porosity, and bioactivity to individual anatomical and biological requirements. Additionally, smart and biodegradable materials, capable of controlled degradation and on-demand drug release, represent a promising avenue to enhance healing while reducing long-term complications. Integration of these innovations into animal model research will accelerate the translational pathways and contribute to the development of next-generation orthopedic implants.

## Figures and Tables

**Table 1:** Comparative characteristics of commonly used animal models in orthopedic implantology. Parameters include osteonal remodeling, skeletal maturity, cartilage thickness, ossification centers, similarity to human bone, healing capacity, tissue integration, vascularization, and ethical considerations. This overview highlights species-specific advantages and limitations relevant to preclinical implant research.

Species	Osteonal Remodeling	Age of Skeletal Maturity (Months/Years)	Average Cartilage Thickness (Femur, mm)	Secondary Centers of Ossification	Macroscopic Similarity to Human Bone	Healing Potential	Tissue Integration Potential	Ethical Considerations
Human	Yes	16–20 years	2.2–2.5	Yes	N/A	Slow–Moderate	High	High [11]
Non-Human Primate	Yes	5–7 years	0.57–0.72	No	Very Similar	Moderate	High	Moderate to High [12]
Rodent	Minimal	26 weeks	0.030 (mouse), −0.17 (rat)	No	Not Similar	Rapid	Low	Low [13]
Rabbit	Minimal (age-dependent)	8–11 months	0.2–0.44	No	Minimally Similar	Moderate	Moderate	Moderate [11]
Dog	Yes	10–18 months	0.6–1.3	No	Moderately Similar	Rapid	High	Moderate to High [11]
Pig	Yes (>6 months)	18–24 months (mini), 2–4 years (conventional)	1.17–1.27	Yes	Moderately Similar	Moderate	Moderate	Moderate [12]
Small Ruminant (Sheep)	Yes (>1 year)	15–18 months (sheep)	0.4–0.5	Yes	Very Similar	Slow to Moderate	High	Moderate [11]
Goat	Yes	2–3 years (goat)	0.7–1.5	Yes	Very Similar	Moderate	High	Moderate [11]

## Data Availability

Data are contained within the article.
